# Assessment of Stress in Dogs Under Cancer Therapy via Faecal Cortisol Metabolite Analysis: A Pilot Study

**DOI:** 10.3390/ani15121809

**Published:** 2025-06-19

**Authors:** Christina Ziegerhofer, Alexander Tichy, Miriam Kleiter, Birgitt Wolfesberger, Rupert Palme

**Affiliations:** 1 Department for Small Animals and Horses, University of Veterinary Medicine Vienna, Veterinaerplatz 1, 1210 Vienna, Austria; miriam.kleiter@vetmeduni.ac.at (M.K.); birgitt.wolfesberger@vetmeduni.ac.at (B.W.); 2 Platform for Bioinformatics and Biostatistics, Department of Biological Sciences and Pathobiology, University of Veterinary Medicine Vienna, Veterinaerplatz 1, 1210 Vienna, Austria; alexander.tichy@vetmeduni.ac.at; 3 Experimental Endocrinology, Department of Biological Sciences and Pathobiology, University of Veterinary Medicine Vienna, Veterinaerplatz 1, 1210 Vienna, Austria; rupert.palme@vetmeduni.ac.at

**Keywords:** stress, canine, chemotherapy, radiation therapy, faeces, glucocorticoid metabolites

## Abstract

Stress reactions play an important role in animals’ ability to cope with various situations. Glucocorticoids are measured as a stress parameter, and analysis of their faecal metabolites has proven to be a good method for evaluating long term stress. We hypothesised that dogs suffering from cancer would have a higher stress level during cancer therapy, which would be reflected in higher levels of faecal cortisol metabolites (FCMs). Faeces were collected from 40 dogs receiving radiation or chemotherapy before and during the treatment, and from 53 healthy dogs, who served as a control group. FCMs were extracted and quantified by a cortisol enzyme immunoassay. The results showed that the stress levels were not significantly higher in the cancer patients before therapy compared to those in the control group. Additionally, there were no significant differences in the FCM concentrations of dogs at specific time points during chemotherapy or radiotherapy. Therefore, suspected stress should not be a criterion to exclude animals from cancer treatment. Such a treatment seems ethically justifiable if it is expected to provide benefits and improvement in the quality of life for patients.

## Simple Summary

The aim of this study was to investigate whether dogs receiving cancer therapy are exposed to additional stress due to their treatment. Thus, faeces from dogs were collected before and during therapy from their owners at home to avoid the stress of visiting the veterinarian at the clinic. Stress hormone metabolites were then measured from the faecal samples. This long-established method has proven to be reliable for non-invasively evaluating stress levels. Our results indicate that tumour therapy did not add to the stress levels of dogs suffering from cancer. This finding could help owners in their decision-making on whether to attempt cancer therapy for their companion dogs.

## 1. Introduction

There is no simple definition of stress in general, but it has been described as a response to any stimulus that threatens or appears to threaten the homeostasis of an individual [1]. Stress involves multiple systems, and can be assumed to constitute allostasis, a term that better characterises stress. Factors such as genetics, the environment and experience fundamentally determine allostasis, and thus the physical and psychological impact of acute and chronic stress [2]. During a stressful situation, a cascade of changes in several systems, like the nervous, endocrine, cardiovascular and immune systems, may be activated in order to help an organism to cope with the situation [3]. An essential part of the stress-response system is the activation of the hypothalamic–pituitary–adrenocortical axis, which elevates glucocorticoid concentrations to mobilise energy for use in fighting the stressor [4,5].

Dogs suffering from cancer are increasingly receiving specific cancer therapy to extend their lifespan and, more importantly, to improve their quality of life. Chemotherapy is the method of choice for primary systemic tumour diseases, such as hematopoietic tumours, but also for inoperable or metastatic solid tumours [6] (p. 98). Radiation therapy is primarily used as a single therapy, especially if the surgical removal of a local tumour is not possible or not entirely possible [6] (p. 132). In addition to chemotherapy, adjuvant radiation therapy can be used to reduce the tumour mass or increase the effectiveness of chemotherapy [6] (pp. 132–134). Many owners fear that frequent oncological treatments are very stressful for their pets. However, scientifically, this has not been proven to be true so far in dogs.

Therefore, the aim of this study was to determine whether the tumour status in dogs is correlated with a higher stress level, and whether cancer therapies cause an increase in stress in dogs. To achieve this aim, a non-invasive method of evaluating adrenocortical activity by analysing faecal cortisol metabolites (FCMs) was selected. Glucocorticoids (mainly cortisol in dogs) are released from the adrenal cortex into the blood stream to allow an animal to cope with stressful situations. They are heavily metabolised, mainly in the liver, and excreted via the bile into the intestine, before finally ending up in the faeces [7,8]. The measurement of FCMs has been described as a useful, non-invasive tool for monitoring stress levels in various animal species [9,10], including various canine species [11,12,13,14,15,16,17,18,19], which is one reason for using this method in this study. Also, this method provides a quantitative and reproducible method for stress assessment. In addition, one study showed that in case of chronic stress, faecal samples are more reliable because they yield a more robust measure that is less affected by short term fluctuations [12]. In general, FCMs reflect cortisol production over a period of about 24 h in dogs, enabling the assessment of chronic stress responses [11].

We hypothesised that dogs with cancer would show higher faecal cortisol metabolite concentrations compared to healthy dogs due to their illness and therapy.

## 2. Materials and Methods

### 2.1. Selection of Dogs for the Study

Client-owned dogs, presented to the University of Veterinary Medicine Vienna between January 2019 and September 2021, who had recently been diagnosed with cancer and were scheduled to receive cancer therapy (radiation therapy with a duration of 2–4 min per session or chemotherapy with a duration of approximately 20–30 min per session) were eligible for the study, regardless of tumour type, age, sex or breed. The study excluded all patients who had previously received or should receive synthetic glucocorticoids as part of the therapy. Healthy dogs that were not clinical patients represented the control group. Therefore, dogs were selected who were not receiving any therapy at the time of sample collection, and who had not had a visit to the vet or experienced any other conscious stressful event in the previous 4 weeks. Attempts were made to keep the sex and breeds approximately similar to those of the cancer patients. Variables regarding age, weight, sex, type of tumours and treatment schedules were recorded. Clinical data were collected using the computer information system of the University of Veterinary Medicine Vienna. Study enrolment required the informed, written consent of owners for the planned investigations.

### 2.2. Description of the Study Participants

In total, 40 dogs were included in the cancer group (15 dogs receiving chemotherapy, 25 dogs receiving radiation therapy), and the results were compared with those for the 53 healthy dogs in the control group. In the control group, the sex distribution was 10 males, 13 neutered males, 8 females and 22 neutered females. The average age was 6.8 years, with a minimum age of 1 year and a maximum age of 16 years, and the average weight was 19.7 kg, with a minimum of 2.2 kg and a maximum of 42.4 kg. With regard to breed, 22 dogs were mixed breeds, 7 dogs were Retrievers (4 Labrador Retrievers and 3 Golden Retrievers), 4 dogs were Chihuahuas and the remaining 20 dogs were of various breeds (Foxterrier, Manchester Terrier, Bolonka Zwetna, Standard Poodle, Brandlbracke, Lagotto Romagnolo, Malinois, Cavalier King Charles Spaniel, American Staffordshire Terrier, Small Münsterländer, Whippet, Boxer, Westhighland White Terrier, Old English Sheepdog, Rhodesian Ridgeback, Jack Russell Terrier and Entlebucher Mountain Dog).

Of the 40 oncologic patients, 8 were males, 5 were neutered males, 4 were females and 23 were neutered females. The average age was 9.1 years, with a minimum age of 4 years and a maximum age of 13 years. The average weight was 26.2 kg, with a minimum of 5 kg and a maximum of 64 kg. With regard to breed, 17 dogs were mixed breeds, 10 dogs were Retrievers (5 Golden Retrievers, 3 Flat-Coated Retrievers and 2 Labrador Retrievers), and the remaining 13 dogs were of various breeds (Parson Russell Terrier, Cane Corso, Magyar Viszla, Maltese, Bernese Mountain Dogs, Australian Shepherd, Bavarian Mountain Hound, Landseer, Bordercollie, Boxer, Beagle and Rhodesian Ridgeback). The most frequently occurring tumour type was sarcoma (18 cases). Six of these cases were located in the skin of the limbs, including liposarcoma, schwannoma, spindle cell sarcoma and soft tissue sarcoma. Seven were osteosarcomas, with five of them located in the bones of the limbs, one located in the nose and one located in the mandible. The remaining sarcoma types were one stickersarcoma located in the vagina, one histiocytic sarcoma located in the spleen, one soft tissue sarcoma located in the mandibulary lymphnode and two hemangiosarcomas located in the spleen. The second most frequently occurring tumour type was carcinoma (14 cases); 3 of these cases were located in the oral cavity, 1 was located in the breast tissue, 3 were located in the urinary bladder, 1 was located in the thyroid gland, 3 were located in the nose, 1 was located in the vagina and 1 was located in the liver. The third most frequent tumour type was melanoma, which affected 6 dogs, with 5 cases located in the oral cavity and 1 located on the front paw. The last two tumour types were a chemodectoma in one dog and a mesothelioma in one dog.

### 2.3. Description of the Treatment Protocols

As tumour treatment, 15 dogs received chemotherapy and 25 dogs received radiation therapy. The administered chemotherapeutic drugs included carboplatin, doxorubicin, mitoxantrone, vincristine and cyclophosphamide. Radiation protocols were divided into palliative protocols, with 5 or 6 daily or once- or twice-weekly radiation sessions, and definite protocols, consisting of 15 or 16 daily sessions.

### 2.4. Faeces Sampling

Patients who received radiation therapy were sampled before the beginning of the therapy and one and two weeks after the start of the therapy. Therefore, one sample was taken on day 0, day 7 and day 14. Patients who received chemotherapy were also sampled before starting therapy, 3 days later, and one, two and three weeks afterwards. So, one sample was taken on day 0, day 3, day 7, day 14 and day 21, respectively. Different collection schemes between radiation patients and chemotherapy patients had to be used, because patients who have been treated with radiation therapy often need a mild dose of synthetic glucocorticoids (prednisolone) after day 14 due to acute side effects. While no apparent side effects were expected in radiation patients on day 3, chemotherapy patients can already begin to suffer in this time period from clinical symptoms such as inappetence, lethargy, vomiting and diarrhoea.

To avoid the inconvenience of visiting the clinic, faecal samples were collected from the dogs at home by their owners. The samples were frozen at −20 °C within one hour after defecation, and they remained frozen during transport to the University with cool boxes and cold packs. After receiving the samples, they were stored in a freezer at the Experimental Endocrinology at the University of Veterinary Medicine in Vienna until analysis.

### 2.5. Analysis of Faecal Cortisol Metabolites (FCMs)

Faecal samples were extracted as previously described by Palme et al. [20]. We suspended 0.5 g of each thawed and well-homogenised faecal sample in 5 mL of 80% methanol (Merck KGaA, Darmstadt, Germany). After shaking for thirty minutes, samples were centrifuged at 2500× *g* for 15 min (AllegraTM X-12R, Beckman Coulter, Krefeld, Germany). Afterwards, a part of the supernatant was diluted (1 + 9) with assay buffer and stored at −20 °C until analysis with a cortisol enzyme immunoassay (EIA) [21]. For details of the EIA, including cross-reactivities, see Palme and Möstl [21]. The sensitivity of the method to measure FCMs was 0.7 ng/g. The intra- and interassay coefficients of variation were below 10 and 15%, respectively. This assay has been successfully validated for measuring faecal cortisol metabolites in dogs to evaluate adrenocortical activity [11].

### 2.6. Statistical Analysis

Data were analysed using IBM SPSS v24. Descriptive statistics are given for both the original cortisol values and the log-transformed values, which were used for the statistical models. The difference in mean cortisol between the control and the cancer group, as well as the difference in mean cortisol between the samples taken before therapy and at specific time points within the radiation and chemotherapy group, were analysed using linear mixed models, followed by post hoc tests for multiple comparisons with Sidak’s alpha correction procedure. In the latter model, the subject was added as a random factor to the model, with a random intercept to estimate the variance explained by the subject. The assumption of normal distribution was assessed by the Kolmogorov–Smirnov test and it was met for the log-transformed values. For all statistical analyses, a *p*-value below 5% (*p* < 0.05) was considered to be significant.

## 3. Results

**Figure 1:**
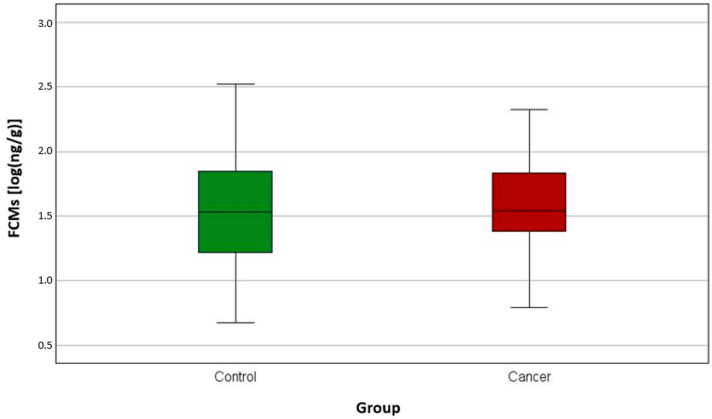
A boxplot of the concentrations [log(ng/g)] of faecal cortisol metabolites (FCMs) in control and cancer dogs at day 0. There was no significant difference between the healthy control group and the cancer-bearing dogs before therapy (*p* = 0.430).

**Figure 2:**
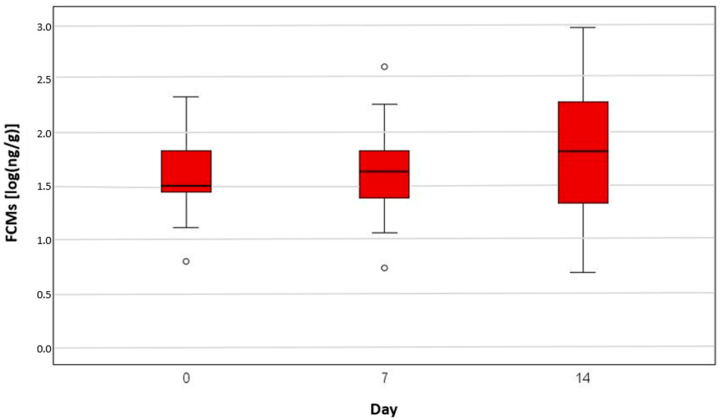
A boxplot faecal cortisol metabolite (FCM) concentrations [log(ng/g)] measured in the radiation group on day 0, 7 and 14. There were no significant differences between the time points (*p* = 0.817 between day 0 and day 7 and *p* = 0.054 between day 0 and day 14).

**Figure 3:**
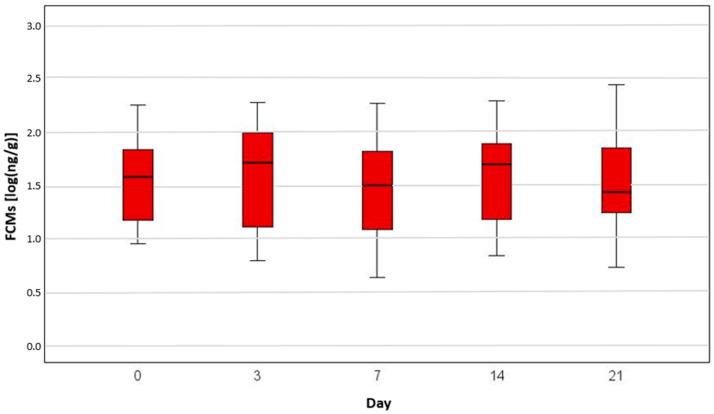
A boxplot of the faecal cortisol metabolite (FCM) concentrations [log(ng/g)] measured in the chemotherapy group on day 0, 3, 7, 14 and 21. There were no significant differences between the time points (*p* = 0.965 between day 0 and day 3, *p* = 0.340 between day 0 and day 7, *p* = 0.934 between day 0 and day 14, and *p* = 0.693 between day 0 and day 21).

We found no significant difference in FCM levels between cancer-bearing dogs before receiving therapy and healthy animals (Figure 1). In addition, there was no significant difference at specific time points during their radiotherapy (Figure 2) or chemotherapy (Figure 3).

The median FCM levels were 33.9 ng/g, 37.0 ng/g and 31.2 ng/g in the control group, chemotherapy group and radiation group, respectively, on day 0. The highest level was measured in one of the radiation-group dogs (933 ng/g on day 14), the second highest was in one of the radiation patients (498 ng/g also on day 14) and the third highest measurement was in one of the healthy dogs (334 ng/g). The lowest stress level was found in two dogs of the healthy group, with 4.7 ng/g and 4.8 ng/g, and in one radiation patient, with 4.9 ng/g, on day 14 (Table 1).

The log-transformed cortisol median value on day 0 for the control group was 1.5 [log(ng/g)], for the chemotherapy group it was 1.6 [log(ng/g)] and for the radiation group it was 1.5 [log(ng/g)] (Table 1).

The cortisol level was not significantly higher in the patients receiving oncological treatment versus the healthy group. Thus, it seems that the tumour-bearing animals were not more stressed than the control group. This assumption can also be made for specific time points for dogs receiving treatments such as chemotherapy or radiation therapy.

## 4. Discussion

To the best of our knowledge, no study has been published on the assessment of stress levels in cancer dogs. Interestingly, we found no significant difference in the levels of cortisol metabolites between treatment-naive dogs diagnosed with tumours and healthy dogs. Thus, we can assume that in these dogs, the cancer status did not cause any additional stress. In addition, we found no significant difference in the levels of cortisol metabolites in the cancer-bearing dogs at different time points during their treatment. Thus, we can assume that the cancer therapy itself did not cause any additional stress. However, we have to consider that besides the cancer therapy itself, additional factors, such as the visit to a veterinarian and the transport to get there (car or public transport), can act as short-term stressors. Also, nutrition and gastrointestinal transit time may have an impact on FCMs, and thus on the interpretation of stress levels in dogs. In one study, the gut microbiome was measured in healthy dogs who were exposed to an acute stressful event like car travel or separation, and this work showed no significant impact on the gut microbiota of dogs [22]. The gut microbiome, dietary composition and individual differences can influence the transit time and FCM concentrations. Therefore, a standardised feeding and controlled sampling time is recommended for future studies to ensure comparability. In human medicine, differences in cortisol concentrations between breast cancer patients and controls vary from non-significant [23] to significant [24]. There are various methods to measure glucocorticoids. Cortisol (or its metabolites) can be measured from hair, milk, saliva, blood, urine and faeces. The choice of sample material depends on which potential stressor is being measured [5,25]. In the case of long-term stress, a faecal sample is more reliable, because it excludes circadian effects and short-term stressors have little influence on the overall result. Short-term stress factors or suddenly expected stressful events should be measured using saliva samples, as these allow for more precise time-based analysis of the stressful event [12]. Cortisol measurements from blood samples follow a circadian rhythm in different species, which must also be taken into account when sampling [26]. Also, salivary samples showed similar results to serum samples in dogs in one study [27]. One study showed the effect of a clinical waiting-room environment as a potential stressor in healthy privately owned dogs, where the dogs in the waiting room showed significantly higher serum cortisol concentrations than the control group, who waited in a garden outside; thus, this study suggests that waiting in a clinical veterinary practice waiting room is a potentially stressful situation for dogs, as indicated by blood serum cortisol concentrations after 20 min [28]. There are many studies that deal with the analysis of FCMs in different species within the canine family [13], such as wolves [14], Mexican wolves [15], coyotes [16], African wild dogs [17], silver foxes [18] and island foxes [19]. There are also studies on FCMs in carnivores in general [29], in dogs and cats [11,30,31] and solely in dogs [32,33]. We chose FCM measurement with EIA in this study as it has already been established as a good non-invasive tool for monitoring potential stressful events in dogs [11]. In veterinary medicine, FCM investigations have already been performed in various situations, although not in the context of cancer. Clinically, painful events such as castration or colic in horses cause increased concentrations of glucocorticoid metabolites in the faeces [34], and this has also been observed after foot injuries in elephants [35]. In one study in which dogs and cats were taken to a veterinarian for their yearly vaccination, FCMs significantly increased after the vaccination in both species [31].

For owners, the notion that therapeutic measures add to the stress level of their ill pet is often frightening. Therefore, sometimes, a potentially beneficial therapy is refused, especially in cancer patients. To objectify these subjective sentiments, we evaluated whether tumour therapy could potentially alter stress levels in cancer-bearing dogs. FCM levels measured at various time points in dogs treated either with chemotherapy or radiotherapy showed no significant differences from the pre-treatment baseline. Thus, it seems likely that cancer treatment is not as stressful for dogs as might be assumed.

There are many studies in human medicine that evaluate cortisol concentrations in relation to mindfulness, psychosocial factors and the prediction of survival in cancer patients undergoing chemotherapy and/or radiation therapy [36,37,38,39,40,41,42]. In addition, cortisol levels and symptoms such as nausea have been investigated during cancer chemotherapy [43,44]. Cortisol in these human studies was, however, measured in the blood [37,39,45] or saliva [23,38,40], and therefore, these results are not comparable to our study’s FCM analyses.

There are limitations to this study, as different tumour entities, various types of chemotherapeutic drugs and diverse radiation protocols were included. Additionally, the two treatment groups were not very large; thus, the small sample size and also the short observation time, as well as the presence of confounding factors, like owner compliance, could potentially have influenced the reliability of the findings. Therefore, the findings of this pilot study need to be confirmed in a larger oncology cohort with more homogeneous treatment groups.

## 5. Conclusions

In this study, the stress levels of dogs diagnosed with cancer were no higher compared to those of their healthy counterparts; also, cancer-specific therapy itself did not seem to put any significant additional stress on canine patients. The method of non-invasive measurement of stress hormone metabolites from faeces was a useful tool for analysis; however, further research will be necessary to validate the results of our pilot study.

## Figures and Tables

**Table 1:** Minimum, maximum and percentiles of faecal cortisol metabolites (FCMs) in the control group, chemotherapy group and radiation group in 2 concentrations (ng/g) and [log(ng/g)].

	Group	Day		Minimum	Maximum	Percentiles		
			N			25	Median	75
**FCMs** **(ng/g)**	**Control**	0	53	4.7	334.0	16.4	33.9	72.2
	**Chemotherapy**	0	15	8.7	174.5	14.5	37.0	68.2
		3	15	6.1	185.0	10.6	50.7	97.0
		7	15	4.2	181.4	8.7	31.1	67.2
		14	15	6.9	192.7	11.5	48.6	95.4
		21	15	5.3	270.1	17.0	26.8	97.9
	**Radiation**	0	25	6.2	211.2	27.4	31.2	95.8
		7	25	5.4	406.1	23.3	42.6	77.9
		14	25	4.9	932.8	21.1	66.7	195.6
**FCMs** **[log(ng/g)]**	**Control**	0	53	0.67	2.52	1.21	1.53	1.86
	**Chemotherapy**	0	15	0.94	2.24	1.16	1.57	1.83
		3	15	0.79	2.27	1.02	1.71	1.99
		7	15	0.63	2.26	0.94	1.49	1.83
		14	15	0.84	2.28	1.06	1.69	1.98
		21	15	0.72	2.43	1.23	1.43	1.99
	**Radiation**	0	25	0.79	2.32	1.44	1.49	1.96
		7	25	0.73	2.61	1.37	1.63	1.89
		14	25	0.69	2.97	1.32	1.82	2.29

## Data Availability

The research data for the presented study are included in this article. For further information, the corresponding authors can be contacted.

## Abbreviations

The following abbreviations are used in this manuscript:

EIA	enzyme immunoassay
FCMs	faecal cortisol metabolites
